# Alpk1 Sensitizes Pancreatic Beta Cells to Cytokine-Induced Apoptosis *via* Upregulating TNF-α Signaling Pathway

**DOI:** 10.3389/fimmu.2021.705751

**Published:** 2021-09-21

**Authors:** Fei Ding, Xi Luo, Yiting Tu, Xianlan Duan, Jia Liu, Lijing Jia, Peilin Zheng

**Affiliations:** ^1^Department of Endocrinology, Shenzhen People’s Hospital, The Second Clinical Medical College of Jinan University, The First Affiliated Hospital of Southern University of Science and Technology, Shenzhen, China; ^2^Department of Neurology, Shenzhen Samii International Medical Center (The Fourth People’s Hospital of Shenzhen), Shenzhen, China; ^3^School of Medicine, Southern University of Science and Technology, Shenzhen, China

**Keywords:** Alpk1, pancreatic beta cells, apoptosis, pro-inflammatory cytokines, TNF-α signaling

## Abstract

Pancreatic beta cell failure is the hallmark of type 1 diabetes (T1D). Recent studies have suggested that pathogen recognizing receptors (PRRs) are involved in the survival, proliferation and function of pancreatic beta cells. So far, little is known about the role of alpha-protein kinase 1 (ALPK1), a newly identified cytosolic PRR specific for ADP-β-D-manno-heptose (ADP-heptose), in beta cell survival. In current study we aimed to fill the knowledge gap by investigating the role of Alpk1 in the apoptosis of MIN6 cells, a murine pancreatic beta cell line. We found that the expression of Alpk1 was significantly elevated in MIN6 cells exposed to pro-inflammatory cytokines, but not to streptozotocin, low-dose or high-dose glucose. Activation of Alpk1 by ADP heptose alone was insufficient to induce beta cell apoptosis. However, it significantly exacerbated cytokine-induced apoptosis in MIN6 cells. Mechanistic investigations showed that Alpk1 activation was potent to further induce the expression of tumor necrosis factor (TNF)-α and Fas after cytokine stimulation, possibly due to enhanced activation of the TIFA/TAK1/NF-κB signaling axis. Treatment of GLP-1 receptor agonist decreased the expression of TNF-α and Fas and improved the survival of beta cells exposed to pro-inflammatory cytokines and ADP heptose. In summary, our data suggest that Alpk1 sensitizes beta cells to cytokine-induced apoptosis by potentiating TNF-α signaling pathway, which may provide novel insight into beta cell failure and T1D development.

## Introduction

Type 1 diabetes (T1D) is an autoimmune disease characterized by the infiltration of inflammatory immune cells into pancreatic islets and progressive destruction of insulin-producing beta cells (1, 2). Both innate and adaptive immunity are involved in the injury of beta cells. To date, a variety of pathogen recognizing receptors (PRRs) have been identified, including C-type lectin receptors (CLRs), nucleotide-binding oligomerization domain (NOD)-like receptors (NLRs), Toll-like receptors (TLRs), and retinoid acid inducible gene I (RIG-I)-like receptors (RLRs) (3). Previous studies have shown that several PRRs are expressed in pancreatic beta cells in both humans and animal models (4, 5). Furthermore, activation of TLR3 or TLR4 induces the apoptosis of beta cells, while TLR9 suppresses the differentiation and function of beta cells (6–8). These findings collectively demonstrate that PRRs are involved in the survival and function of pancreatic beta cells.

Alpha-kinase 1 (ALPK1, also known lymphocyte α-kinase) has been recently identified as a new cytosolic PRR specific for ADP-β-D-manno-heptose (ADP-heptose), a metabolite in the biosynthesis of Lipopolysaccharide (LPS) (9). A series of studies have demonstrated that ALPK1 is an upstream kinase to induce the phosphorylation and oligomerization of RAF-interacting protein with forkhead-associated domain (TIFA), and subsequently TGF-β-activated kinase 1 (TAK1) phosphorylation, nuclear factor kappa B (NF-κB) activation and pro-inflammatory cytokine production (10–13), suggesting that ALPK1 is an essential player in inflammation and innate immune responses.

So far, the role of ALPK1 in beta cell survival and function is little known. A recent paper has shown that *Alpk1*-overexpressed C57BL/6 mice exhibited a decreased insulin level and severe hyperglycaemia than wild type mice after streptozotocin (STZ) treatment (13), suggesting that Alpk1 might participate in the destruction of pancreatic beta cells. Therefore, in this study we investigated the role of Alpk1 in the injury of MIN6 cells, a murine pancreatic beta cell line.

## Materials and Methods

### Cell Culture and Treatment

MIN6 cells were maintained in Dulbecco’s modified Eagle’s medium (DMEM, Gibco) supplemented with 15% heat-inactivated fetal bovine serum (FBS, Gibco), 100 U/ml penicillin,100 U/ml streptomycin (Gibco), and 50 µM β-mercaptoethanol (Gibco). Cells were cultured at 37 °C in a humidified atmosphere of 5% CO_2_. MIN6 cells were exposed to a mix of pro-inflammatory cytokines including 10 ng/ml interleukin (IL)-1β, 10 ng/ml tumor necrosis factor (TNF)-α, and 10 ng/ml γ-interferon (IFN-γ) (hereafter referred to as cyto mix), or 10 mM STZ, or 32 µM ADP heptose, or various concentrations of glucose (5.5, 11.1, 25, 33.3 mM) for indicated durations. In some experiments, cells were simultaneously treated with cyto mix and ADP heptose at aforementioned concentrations.

### Chemicals

Recombinant murine IL-1β, IFN-γ, and TNF-α were purchased from Peprotech. ADP heptose (>95% purity) was obtained from J&K Scientific. STZ was purchased from Macklin. GLP-1-receptor-agonist liraglutide (Victoza) was from Novo Nordisk.

### Quantitative Reverse Transcription Polymerase Chain Reaction (qRT-PCR)

Total RNA from the cells was extracted using Direct-zol RNA MiniPrep Plus kit (Zymo Research), and cDNA was synthesized from 1 μg of total RNA by PrimeScript reverse reaction kit, according to the manufacturer’s protocols (Takara). qRT-PCR analysis of Alpk1, TNF-α, IL-1β, and IFN-γ was performed in a StepOne Plus PCR system (Applied Biosystems). The gene expression levels were quantified as a fold change against β-actin by using the 2^-ΔΔCT^ method (14). The following primers were used: mouse *Alpk1* (forward: 5’ -CAGGTTCACGGATGTGACCA-3’, reverse: 5’-GCCCTGTGCATATTTCAGCG-3’); mouse *IFN-γ* (forward: 5’- TCAAGTGGCATAGATGTGGAAGAA-3’, reverse: 5’- TGGCTCTGCAGGATTTTCATG-3’); mouse *Fas* (forward: 5’- AGCCCGTTGGAGTGATTCAA-3’, reverse: 5’- CCCCCTGCAATTTCCGTTTG-3’); mouse TNF-α (forward: 5’- AATGGCCTCCCTCTCATCAGT-3’, reverse: 5’- GCTACAGGCTTGTCACTCGAATT-3’); mouse IL-1β (forward: 5’-TGCCACCTTTTGACAGTGATG-3’, reverse: 5’- TGTGCTGCTGCGAGATTTG-3’); mouse β-actin (forward: 5’- CCCAGCACAATGAAGATCAAGATCAT -3’, reverse: 5’- ATCTGCTGGAAGGTGGACA -3’) (15).

### Western Blotting

Cells were lysed using SDS lysis buffer containing protease and phosphatase inhibitors. The anti-mouse Alpk1 antibody (1:1000) was from Proteintech. The anti-mouse TNF-α, Fas and specificity protein 1 (SP1) antibodies (all 1:500) were purchased from Beyotime. The anti-cleaved Caspase 3 (1:1000), anti-NF-κB P65 (1:1000), anti-tubulin (1:2000) antibodies were from Cell Signaling Technology. The anti-TNF receptor associated factor (TRAF) 2 (1:1000), anti-TRAF6 (1:1000), anti-p-NF-κB P65 (S536, 1:1000), anti-p-TAK1 (S412, 1:1000), anti-TAK1 (1:1000) antibodies were from ABclonal. The anti-p-TIFA (T9, 1:1000), anti-TIFA (1:1000) antibodies were from Abcam.

### Apoptosis Assay

For apoptosis assessment, 1x10^5^ MIN6 cells were plated and treated with cyto mix, or ADP heptose, or cyto mix together with ADP heptose for 24 hours. The dead cells were examined by addition of CellTox Green Cytotoxicity dye. The plate was read on a SPARK 10M reader (TECAN). In some experiments, cells were treated and then stained with Annexin V/PI solution (RiboBio) and measured on a DxFLEX flow cytometer (Beckman Coulter). Data were analyzed with Flowjo version 10.0.7 (Treestar). For TUNEL staining, 1x10^5^ MIN6 cells were plated onto coverslips in 24-well culture plates and treated. A riboAPO One-Step TUNEL Apoptosis Kit (RiboBio) was used to detect DNA fragmentation in cells according to the manufacturer’s instructions. The nuclei were stained with DAPI. TUNEL staining was evaluated by a fluorescence microscopy (Leica TCS SP8). Cells double labeled with DAPI and TUNEL in the nuclei were considered as dead cells.

### Cell Viability Assay

Cell viability was assessed by a CCK-8 kit (Med Chem Express). Briefly, 1x10^5^ MIN6 cells were incubated with ADP heptose, or cyto mix, or cyto mix plus ADP heptose for 24 hours. CCK-8 solution was added and OD (450 nm) was measured on a SPARK 10M reader (TECAN).

### EdU Cell Proliferation Assay

The proliferation of MIN6 cells was assessed by a Cell-Light EdU Kit (RiboBio) according to manufacture instructions. The nuclei were stained with DAPI. EdU incorporation was evaluated by a fluorescence microscopy (Leica TCS SP8). Cells double labeled with DAPI and EdU in the nuclei were considered as dividing cells.

### RNA-Seq Analysis

RNA-Seq analysis was performed by BGI-Shenzhen. Briefly, total RNA was extracted from MIN6 cells treated with cyto mix alone or together with ADP heptose for 24 hours using Trizol (Invitrogen) according to manual instruction. RNA was qualified and quantified using a Nano Drop and Agilent 2100 bioanalyzer (Thermo Fisher Scientific). Oligo (dT)-attached magnetic beads were used to enrich mRNA, which subsequently fragmented into small pieces. The First-strand cDNA was generated using random hexamer-primed reverse transcription, followed by a second-strand cDNA synthesis and addition of A-Tailing Mix and RNA Index Adapters. The cDNA fragments were validated on the Agilent Technologies 2100 bioanalyzer, and then denatured and circularized into single strand circle DNA (ssCir DNA). The final library was amplified with phi29 to make DNA nanoball (DNB) which had more than 300 copies of one molecular, DNBs were loaded into the patterned nanoarray and single end 50 bases reads were generated on BGIseq500 platform. The original sequencing data were deposited to Sequence Read Archive (SRA) with the accession number PRJNA726429 (https://www.ncbi.nlm.nih.gov/bioproject/PRJNA726429).

### Isolation of Nuclear Proteins

Nuclear proteins of MIN6 cells were isolated according to manufacturer’s instructions (Thermo Scientific, subcellular protein fractionation kit for cultured cells). Briefly, MIN6 cells were treated and washed twice with ice-cold PBS. 5×10^6^ cells were then to a 1.5mL microcentrifuge tube for nuclear protein isolation.

### Statistical Analysis

Statistical analyses were performed with Prism version 8.3.0 (GraphPad). Data were analyzed with unpaired Student’s t-test (two-tailed) or one-way analysis of variance followed by Tukey’s *post hoc* test. *p*<0.05 was considered statistical significant.

## Results

### Pro-Inflammatory Cytokines Induced the Expression of Alpk1 in MIN6 Cells

**Figure 1:**
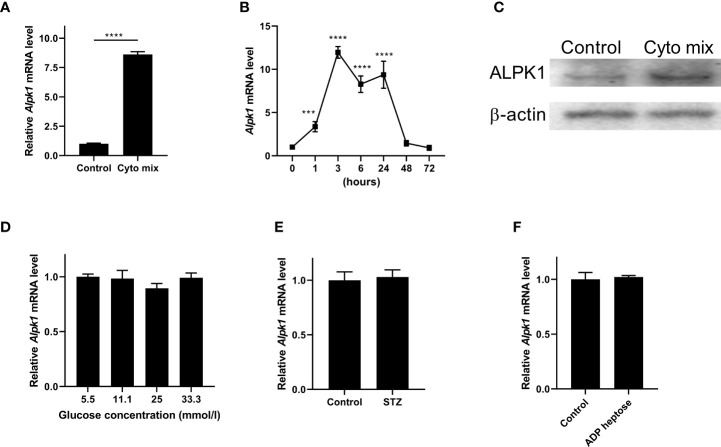
Alpk1 expression was induced by pro-inflammatory cytokines in MIN6 cells. **(A)** MIN6 cells were untreated or treated with a mix of pro-inflammatory cytokines (cyto mix; 10 ng/ml IL-1β, 10 ng/ml TNF-α, and 10 ng/ml IFN-γ) for 24 hours. *Alpk1* mRNA level was measured by qRT-PCR. **(B)** Time-course expression of *Alpk1* exposed to cyto mix. **(C)** MIN6 cells were untreated or treated with cyto mix for 24 hours. Alpk1 protein level was measured by western blotting. **(D)** MIN6 cells were treated with various concentrations of glucose for 24 hours. **(E)** MIN6 cells were treated with vehicle or 10 mM STZ for 24 hours. **(F)** MIN6 cells were treated with vehicle or 32 µM ADP heptose for 24 hours. *Alpk1* mRNA level was measured by qRT-PCR. Data show mean ± SD and are representative of 3 independent experiments. The data shown in **(A, E, F)** are were analyzed with unpaired Student’s t-test, and data in **(B, D)** were analyzed with ANOVA followed by Tukey’s *post hoc* test. ***P < 0.001; ****P < 0.0001. Cyto mix, a mix of pro-inflammatory cytokines; STZ, streptozotocin.

Pro-inflammatory cytokines such as IFN-γ, TNF-α, and IL-1β, STZ and glucose toxicity play essential roles in the failure of pancreatic beta cells (1, 16, 17). To investigate the role of Alpk1 in beta cell injury, we first used cyto mix (10 ng/ml IL-1β, 10 ng/ml TNF-α, and 10 ng/ml IFN-γ), or various concentrations of glucose, or STZ to treat MIN6 cells, and examined the expression of Alpk1 by qRT-PCR. The mRNA level of *Alpk1* was significantly induced when cells were exposed to cyto mix for 24 hours (**Figure 1A**). Time-course profiling showed that following cyto mix treatment, *Alpk1* mRNA level was significantly increased even at 1 hour, maintained at a nearly 10-fold increase from 3 to 24 hours, and then decreased to baseline at 48 and 72 hours (**Figure 1B**). Correspondingly, Alpk1 protein was significantly elevated in response to cyto mix at 24 hours (**Figure 1C**). No alterations of *Alpk1* expression were observed in cells treated with low or high concentrations of glucose, or STZ (**Figures 1D, E** **)**. Activation of Alpk1 by ADP heptose also did not affect *Alpk1* expression (**Figure 1F**). Collectively, these data indicated that Alpk1 might be involved in cytokine-induced beta cell injury.

### Alpk1 Activation by ADP Heptose Impaired the Viability of Cytokine-Treated MIN6 Cells

**Figure 2:**
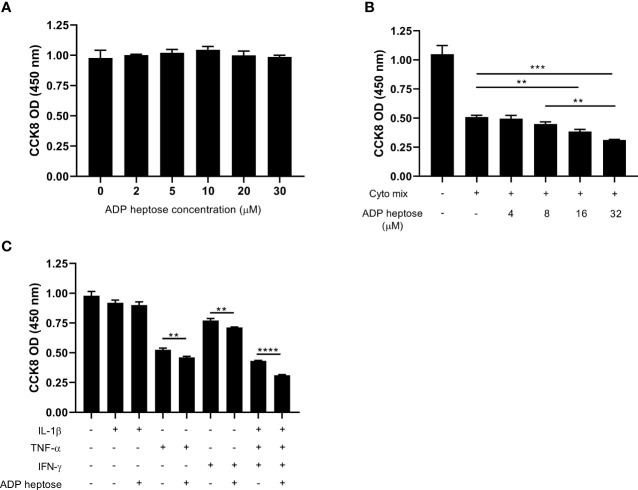
Alpk1 activation synergized with cyto mix to reduce cell viability. The number of viable cells were assayed by CCK-8 solution. **(A)** 1x10^5^ MIN6 cells were treated with vehicle (sterile distilled water), or a series concentration of ADP heptose for 24 hours. **(B)** MIN6 cells were treated with vehicle (sterile distilled water), or cyto mix, or cyto mix together with elevated concentrations of ADP heptose for 24 hours. **(C)** MIN6 cells were treated with the indicated cytokine with or without ADP heptose (32 µM) for 24 hours. Data show mean ± SD and are representative of 3 independent experiments. The data shown in **(A, B)** are were analyzed with ANOVA followed by Tukey’s *post hoc* test, and data in **(C)** were analyzed with unpaired Student’s t-test. **P < 0.01; ***P < 0.001; ****P < 0.0001.

ALPK1 is a PRR that can be activated by its agonist, ADP heptose. We next investigated whether ADP-heptose-induced Alpk1 activation impaired beta cell survival. A little surprisingly, the viability of MIN6 cells was not affected when exposed to a series concentration of ADP heptose (**Figure 2A**). Considering Alpk1 expression was induced by cyto mix and both pro-inflammatory cytokines and PRR agonists were elevated in the sera of T1D patients (18, 19), we investigated the combinational effects of cyto mix and Alpk1 activation on beta cell survival. Cyto mix impaired beta cell viability. Of note, Alpk1 activation exacerbated cytokine-induced beta cell death in a dose dependent manner (**Figure 2B**). We further determined which component of cyto mix were synergized with Alpk1. It was found that two cytokines, TNF-α and IFN-γ, could synergize with Alpk1 to reduce beta cell viability (**Figure 2C**).

### Alpk1 Activation by ADP Heptose Exacerbated Cytokine-Induced Beta Cell Death

**Figure 3:**
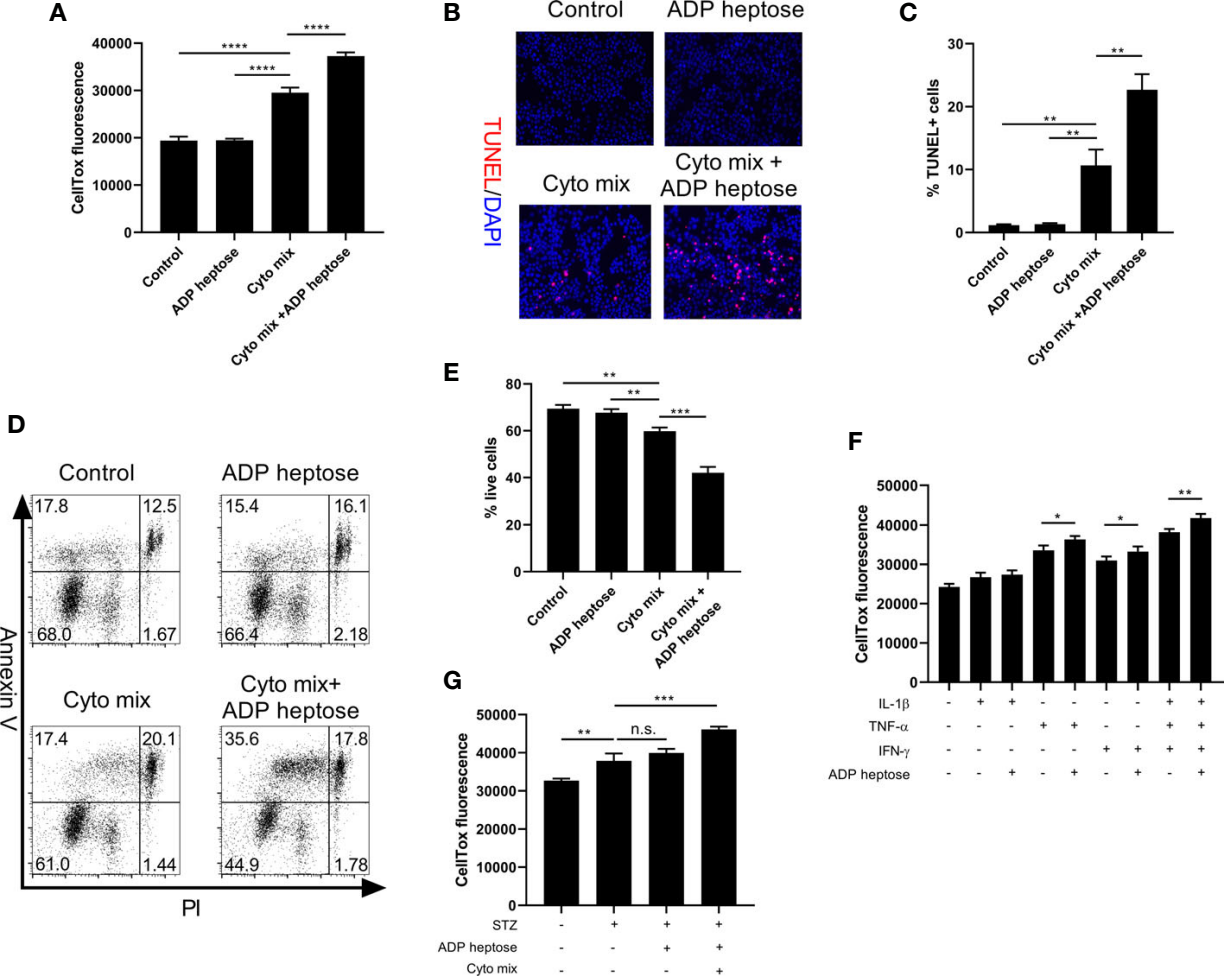
Alpk1 activation exacerbated cytokine-induced beta cell death. 1x10^5^ MIN6 cells were treated with vehicle, or ADP heptose (32 µM), or cyto mix, or cyto mix plus ADP heptose (32 µM) for 24 hours. **(A)** The death of MIN6 cells as assayed by the staining of CellTox Dye. **(B)** Representative images with at 100X magnification showing apoptotic MIN6 cells (TUNEL^+^DAPI^+^) with summary **(C)**. Red, TUNEL; Blue, DAPI. **(D)** Representative flow plots showing Annexin V/PI staining of MIN6 cells with summary **(E)**. **(F)** The death of MIN6 cells exposed to individual cytokine with or without ADP heptose, as assayed by the staining of CellTox Dye. **(G)** The death of MIN6 cells exposed to vehicle, or STZ, or STZ and ADP heptose, or STZ, ADP heptose and cyto mix, as assayed by the staining of CellTox Dye. Data show mean ± SD and are representative of 3 independent experiments. Data were analyzed with unpaired Student’s t-test. **P < 0.01; ***P < 0.001; ****P < 0.0001. TUNEL, Terminal deoxynucleotidyl transferase dUTP nick end labeling; DAPI, 4’,6-diamidino-2-phenylindole; PI, Propidium Iodide; n.s., not significant.

We proceeded to ask whether the decreased viable cell number was due to suppressed proliferation or enhanced apoptosis after cyto mix and ADP heptose treatment. We found that activation of Alpk1 by ADP heptose did not alter cell proliferation (**Supplementary Figures 1A, B** **)**. In contrast, Alpk1 activation significantly exacerbated apoptosis in cyto mix-treated beta cells, as revealed by the staining of a dead cell dye (**Figure 3A**), terminal deoxynucleotidyl transferase dUTP nick end labeling (TUNEL, **Figures 3B, C** **)** and Annexin V/Propidium Iodide (**Figures 3D, E** **)**. We also evaluated the effects of ALPK1 activation on the apoptosis of cells treated with individual cytokine. Consistent with the cell viability data, Alpk1 activation could synergize with TNF-α or IFN-γ to induce more cell death (**Figure 3F**). Although STZ exerted little effect on the expression level of *Alpk1* in MIN6 cells, we further investigated whether these two could be functional synergistic. Intriguingly, the addition of ADP heptose did not alter the apoptosis of STZ-treated cells (**Figure 3G**).

### ADP-Heptose-Induced Alpk1 Activation Enhanced TNF-α Signaling Pathway in Cytokine-Treated Beta Cells

**Figure 4:**
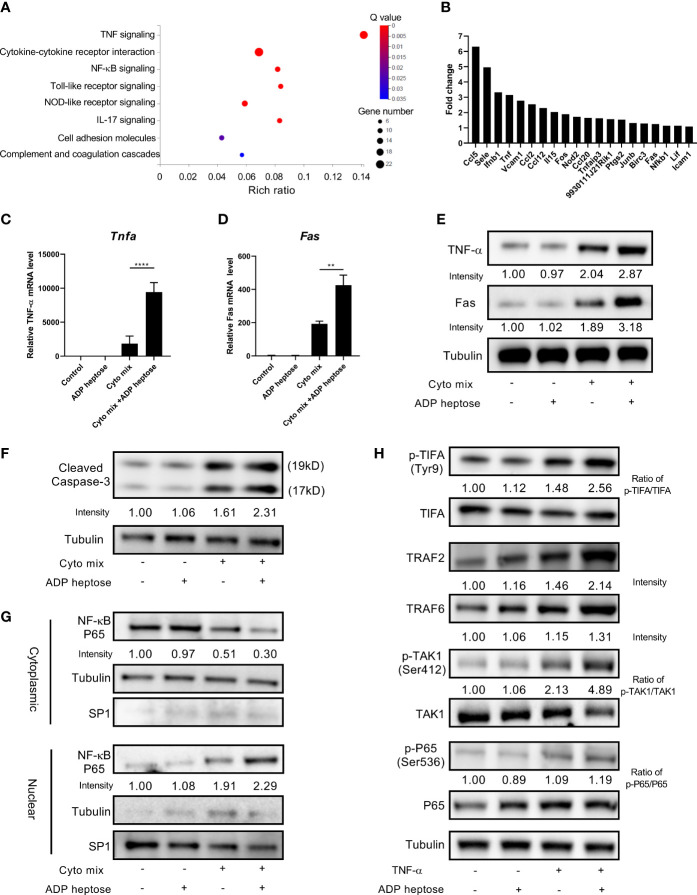
ADP-heptose-induced ALPK1 activation enhanced TNF-α signaling pathways in cytokine-treated beta cells. MIN6 cells were treated with cyto mix or cyto mix plus ADP heptose for 24 hours. RNA from treated cells were extracted and sequenced. **(A)** Bubble chart showing the significantly enriched KEGG pathways for differentially expressed genes in MIN6 cells simultaneously treated with cyto mix and ADP heptose (Q value < 0.05). The size of the bubble represents the number of genes annotated to KEGG Pathway. The color represents the enriched significance. The redder the color, the smaller the significance value. **(B)** The expression fold change of twenty differentially expressed genes in enriched TNF-α signaling pathway. MIN6 cells were treated with vehicle, or ADP heptose (32 µM), or cyto mix, or cyto mix plus ADP heptose (32 µM) for 24 hours. *TNF-α* **(C)** and *Fas* **(D)** mRNA level was measured by qRT-PCR. **(E)** TNF-α and Fas protein levels were examined by western blotting. **(F)** The level of cleaved Caspase-3 was examined by western blotting. **(G)** The nuclear proteins of MIN6 cells were fractioned by subcellular protein fractionation kit for cultured cells (Thermo Scientific, #78840). The levels of cytoplasmic and nuclear NF-κB P65 were detected by western blotting. The cytoplasmic protein Tubulin and nuclear protein SP1 were served as the internal control. **(H)** MIN6 cells were treated with 10 ng/ml TNF-α, and or 32 µM ADP heptose for 4 hours. Cells were then lysed in lysis buffer containing phosphatase and proteinase inhibitors. Data show mean ± SD and are pooled from 3 independent experiments. The data shown in **(C, D)** were analyzed with unpaired Student’s t-test. **P < 0.01; ****P < 0.0001.

Since we demonstrated that Alpk1 activation sensitized pancreatic beta cells to cytokine-induced apoptosis by various approaches, we next investigated the potential mechanism by RNA-Seq analysis. RNA from MIN6 cells exposed to cyto mix alone or cyto mix together with ADP heptose were extracted and sequenced. Notably, we found that TNF-α signaling pathway was the most enriched pathway for differentially expressed genes (DEGs) in cells simultaneously treated with cyto mix and ADP heptose (**Figure 4A**). Two upstream factors, TNF-α and Fas, were significantly upregulated among 20 DEGs in enriched TNF-α signaling pathway (**Figure 4B**). The elevated expression of TNF-α and Fas were further confirmed in separate experiments by qRT-PCR and western blotting (**Figures 4C–E**). We also investigated whether Alpk1 activation induced the expression of IL-1β and IFN-γ, which were the other two components in cyto mix. Interestingly, *IL-1β* and *IFN-γ* mRNA levels were comparable in cytokine-treated cells with or without ADP heptose (data not shown). Caspase-3 is one of essential downstream effectors in TNF-α signaling pathway. Western blotting analysis showed that the amount of cleaved caspase-3 was elevated in cells treated with cyto mix, and was further increased in the presence of cyto mix and ADP heptose (**Figure 4F**). Consistently, the abundance of nuclear NF-κB, a pivotal transcription factor in TNF-α signaling pathway, was most increased when cells were exposed to cyto mix and ADP heptose (**Figure 4G**). To further investigate the possible underlying molecular mechanism for the synergistic effect of ALPK1 and TNF-α, we evaluated the activation of key molecules in the ALPK1 and TNF-α signaling pathways. Intriguingly, TNF-α exposure in MIN6 cells was sufficient to phosphorylate and activate TIFA, a direct downstream effector of ALPK1. The addition of ADP heptose further enhanced TIFA activation, upregulated the expression of TIFAsome components such as TRAF2 and TRAF6, and subsequently induced an increased phosphorylation of TAK1 and NF-κB P65 (**Figure 4H**).

### Glucagon-Like Peptide-1 Receptor (GLP-1R) Agonist Ameliorated Apoptosis in Beta Cells Exposed to ADP Heptose and Cyto Mix

**Figure 5:**
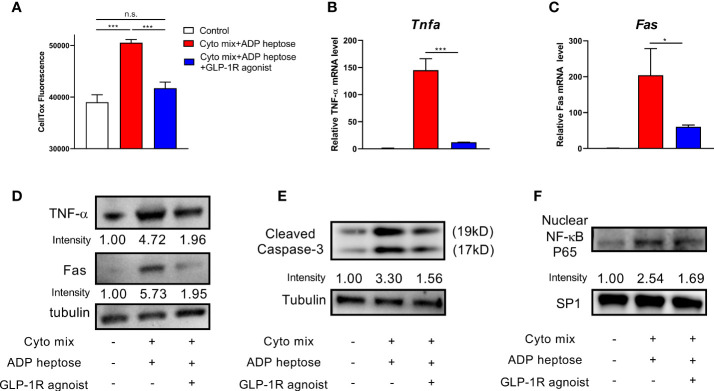
GLP-1R agonist ameliorated apoptosis in beta cells exposed to ADP heptose and cyto mix. **(A)** The death of MIN6 cells as assayed by the staining of CellTox Dye. The changes of mRNA **(B, C)** and protein **(D)** levels of TNF-α and Fas when cyto mix and ADP heptose treated MIN6 cells exposed GLP-1R agonist (100 nM) for 24 hours. **(E)** The level of cleaved Caspase-3 was examined by western blotting. **(F)** The nuclear proteins of MIN6 cells were fractioned, and the level of NF-κB P65 was detected by western blotting. The nuclear protein SP1 was served as the internal control. Data show mean ± SD and are representative of 3 independent experiments. The data shown in **(A–C)** were analyzed with unpaired Student’s t-test *P < 0.05; ***P < 0.001; n.s., not significant.

A previous study have shown that GLP-1R agonist could improve pancreatic beta cell survival when exposed to endoplasmic reticulum stress (20). Therefore, we investigated whether GLP-1R agonist could improve beta cell survival in Alpk1 and cytokine-induced inflammation. We found that GLP-1R activation significantly diminished the apoptosis of cells treated with ADP heptose and cyto mix (**Figure 5A**). The mRNA and protein levels of TNF-α and Fas were significantly reduced (**Figures 5B–D**). The activation of Caspase-3 (**Figure 5E**) and NF-κB (**Figure 5F**) was also dramatically decreased after GLP-1R agonist treatment.

## Discussion

In this study, we provide the first evidence that Alpk1, a newly identified PRR, is involved in the survival of pancreatic beta cells. We showed that pro-inflammatory cytokines, but not STZ, glucose toxicity and ADP heptose, induced Alpk1 expression in pancreatic beta cells. In turn, activation of Alpk1 further sensitized beta cells to cytokine-induced apoptosis, possibly by selectively up-regulating the expression of TNF-α and Fas to elicit a more profound Caspase-3 activation and NF-κB nuclear translocation. Finally, GLP-1R agonist could inhibit TNF-α signaling pathway, and ameliorated the apoptosis of cells exposed to cyto mix and ADP heptose. Our results thus demonstrated a novel link between Alpk1 and TNF-α signaling pathway in beta cell injury.

Previous studies mainly focused on the function of PRRs on immune cells in T1D studies. TLRs constitute a major class of PRRs. The role of TLRs in immune regulation and T1D development has been uncovered by several studies. Deletion of *Tlr7* protected NOD mice from T1D, which might be due to the altered differentiation and reduced antigen-presenting functions of B cells (21). *Tlr9* deficient NOD mice exhibited a decreased incidence of T1D. The disease protection could be imparted by enhanced CD73 expression in T cells and impaired IFNα expression in dendritic cells (22, 23). Of note, specific knockout of *Tlr9* in B cells of NOD mice replicated disease protection, possibly mediated by B cell hyporesponsiveness and elevated IL-10-producing B cells (24). Recent studies have demonstrated that in addition to the dysfunction of immunity, the injury of β cells also plays an essential role in the pathogenesis of T1D (15, 25). So far, the studies of PRRs in pancreatic beta cells are limited. *Tlr9* deficiency in NOD mice did not alter beta cell death, but enhanced CD140a expression and subsequently increased beta cell proliferation and mass (9). Activation of TLR4 by LPS led to enhanced expression of pro-inflammatory cytokines and chemokines, as well as elevated apoptosis in both human and murine pancreatic beta cells (7, 19, 26). Quite differently, in current study we found that activation of Alpk1 alone was insufficient to induce the apoptosis of MIN6 cells. However, Alpk1 activation could exacerbate beta cell death in the presence of pro-inflammatory cytokines. So far we don’t know the underlying mechanism for this distinction. A possible explanation may be that TLR4 is located on the plasma cell surface to form the first line to sense pathogen and counteract infection. On the contrary, Alpk1 is a cytosolic PRR, which might form the second line to amplify danger signals and trigger a more severe inflammation in pancreatic beta cells. Another possible explanation can be that in the resting condition Alpk1 expression is pretty low in MIN6 cells (cycle threshold value >32).Therefore, ADP-heptose-induced Alpk1 activation may be insufficient to trigger inflammatory responses. Cyto mix significantly induced Alpk1 expression, which could promote Alpk1 activation and the downstream inflammatory pathways.

Intriguingly, Alpk1 activation could synergize with TNF-α or IFN-γ, but not IL-1β, to induce more cell death. It has been well documented that NF-κB predominantly mediates IL-1β as well as ALPK1 signaling pathways (10, 27, 28). In contrast, transcription factors such as NF-κB, activating transcription factor 2 (ATF2), C/EBP Homologous Protein (CHOP), cAMP Response Element-Binding Protein (CREB) and ETS Like-1 (ELK1) are involved in TNF-α signaling pathway, while interferon regulatory factor 1 (IRF-1) and signal transducer and activator of transcription 1 (STAT1) participates in IFN-γ mediated signaling transduction (29, 30). Therefore, a possible explanation can be that the excess usage of NF-κB in cells treated with both IL-1β and ADP heptose might hinder their potential synergic effects. Of note, we found that TNF-α treatment was capable of activating TIFA in MIN6 cells, which was consistent with a previous observation that TNF-α stimulation induced TIFA phosphorylation in 293T cells (31). In contrast, ADP heptose stimulation slightly increased TIFA phosphorylation, which might explain why ADP heptose alone had a minimal effect on the apoptosis of MIN6 cells.

Multiple injections of low-dose STZ (MLDS) can cause a low-grade injury of pancreatic beta cells accompanied by elevated inflammation and consequently apoptosis of beta cells (32). A previous study have showed that C57BL/6 mice with *Alpk1* overexpression exhibited normal blood glucose level. After MLDS treatment, those mice showed a lower level of insulin and severe hyperglycemia (13). In line with this observation, we found that Alpk1 activation alone did not alter the proliferation or apoptosis of MIN6 cells. In the presence of inflammation, activation of Alpk1 enhanced the expression of TNF-α and Fas, and might subsequently exacerbate beta cell death. A previous study have showed that treatment of high-dose glucose (200 mg/dL) for 48 hours significantly elevated the expression of Alpk1 in THP1 and HK2 cells (33). However, in this study we found that Alpk1 expression was selectively induced by pro-inflammatory cytokines. It seems that Alpk1 expression can be indeed induced under stress conditions, but may depend on specific cell types or stress.

ADP heptose is an intermediate for the biogenesis of LPS in gram negative bacteria (34). In this study we used a synthesized ADP heptose to investigate the effects of Alpk1 activation on beta cell death *in vitro*. Several studies have illustrated the importance of gut microbiome in the pathogenesis of T1D. The increased intestinal permeability, which precedes the onset of disease, is observed in both T1D patients and animal models (35, 36). The altered permeability of intestinal barriers allows gut bacteria translocating into pancreatic lymph nodes and trigger T1D onset (37–39). Therefore, we speculate that *in vivo* ADP heptose might be derived from microbiome in the leaky gut and contribute to T1D development, which requires further studies.

In current study we used a mix of pro-inflammatory cytokines to induce the injury of pancreatic beta cells, which is more relevant to the pathology of T1D (17). A series of studies have demonstrated that a chronic and low-grade inflammation, characterized by the elevated pro-inflammatory cytokines such as TNF-α and IL-6, exists in obese and type 2 diabetic patients (40, 41). Intriguingly, *ALPK1* variant was reported to be associated with type 2 diabetes (42, 43), suggesting that ALPK1 might also be involved in the injury of pancreatic beta cells in the scenario of type 2 diabetes.

The limitation of the present study is the lack of *in vivo* validation for the role of Alpk1 on beta cell survival. Considering that Alpk1 is pleiotropic and impacts multiple cell types, transgenic mice with beta cell-specific deletion of *Alpk1* may contribute to solve this issue. In conclusion, our data suggest that Alpk1 can sensitize pancreatic beta cells to cytokine-induced apoptosis by upregulating TNF-α signaling pathway. Inhibition of Alpk1 might delay beta cell failure and be an important therapeutic approach for T1D.

## Data Availability Statement

The raw sequencing data have been uploaded and released on BioProject - accession number PRJNA726429 (https://www.ncbi.nlm.nih.gov/bioproject/PRJNA726429).

## Author Contributions

FD, XL, YT, and PZ designed the experiments. XL, FD, XD, and JL conducted the experiments. FD, XL, FD, YT, XD, LJ, and PZ analyzed data. PZ and LJ supervised the study. YT and PZ wrote the manuscript. This project was conceived by PZ who assumes responsibility for the work. All authors contributed to the article and approved the submitted version.

## Funding

This work was funded by the Natural science foundation of Hunan province (2019JJ30036), Natural science foundation of Guangdong province (2020A1515010085), Shenzhen Municipal Science and Technology Innovation Committee Project (JCYJ20190807150619047).

## Conflict of Interest

The authors declare that the research was conducted in the absence of any commercial or financial relationships that could be construed as a potential conflict of interest.

## Publisher’s Note

All claims expressed in this article are solely those of the authors and do not necessarily represent those of their affiliated organizations, or those of the publisher, the editors and the reviewers. Any product that may be evaluated in this article, or claim that may be made by its manufacturer, is not guaranteed or endorsed by the publisher.
