# Supplementary material for: Alpk1 Sensitizes Pancreatic Beta Cells to Cytokine-Induced Apoptosis via Upregulating TNF-α Signaling Pathway
Source: Front Immunol. 2021 Sep 21;12:705751. doi: 10.3389/fimmu.2021.705751 (PMC8490819; doi:10.3389/fimmu.2021.705751)
Supplement: Supplementary file 1 [file DataSheet_1.docx]

Supplementary Material


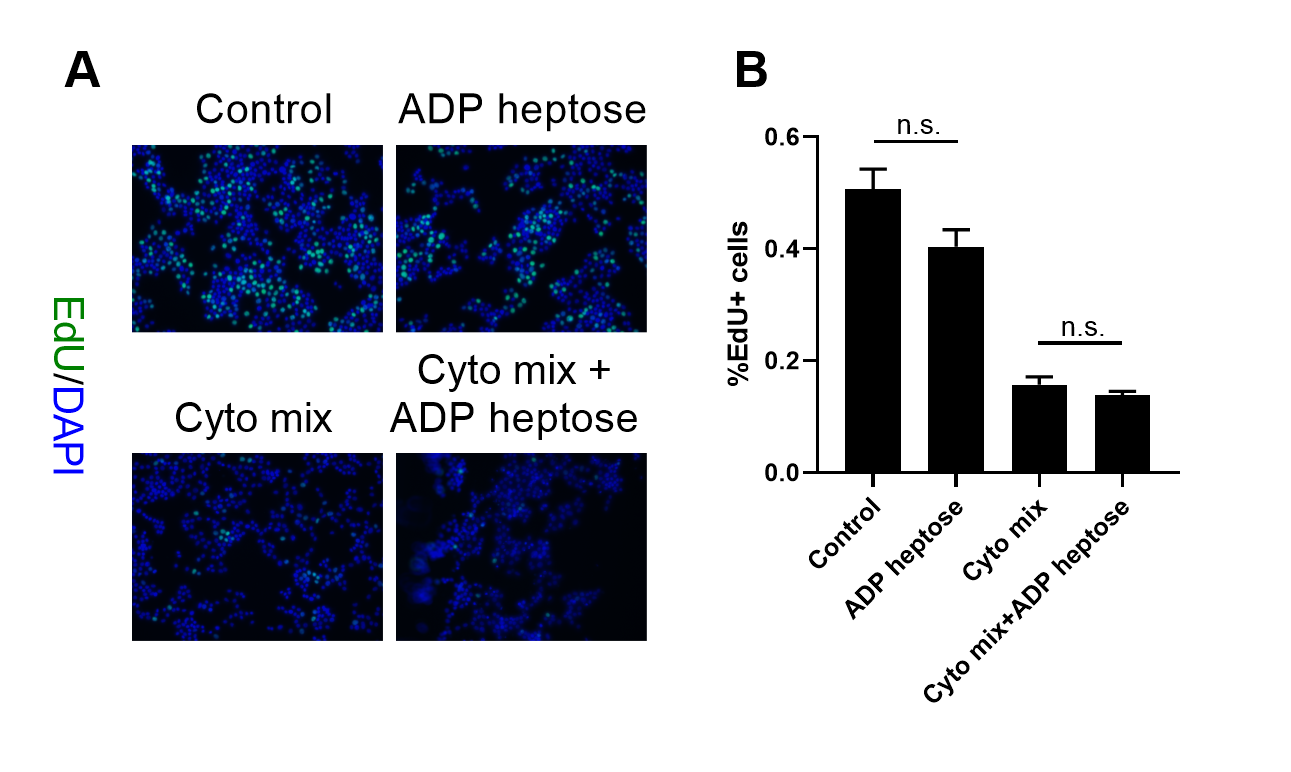


**Supplementary Figure 1.** Alpk1 activation did not alter the proliferation of MIN6 cells. MIN6 cells were treated with vehicle, or ADP heptose (32 µM), or cyto mix, or cyto mix plus ADP heptose (32 µM) for 24 hours. (A) Representative images with at 100X magnification showing proliferating MIN6 cells (EdU+DAPI+) with summary (B). Green, EdU; Blue, DAPI. Data show mean ± SD and are representative of 3 independent experiments.
